# Exploring the dynamics of COVID-19 in a Greenlandic cohort: Mild acute illness and moderate risk of long COVID

**DOI:** 10.1016/j.ijregi.2024.100366

**Published:** 2024-04-14

**Authors:** Mie Møller, Trine Abelsen, Anna Irene Vedel Sørensen, Mikael Andersson, Lennart Friis Hansen, Christine Dilling-Hansen, Nikolai Kirkby, Peter Vedsted, Kåre Mølbak, Anders Koch

**Affiliations:** 1 Greenland Center for Health Research, University of Greenland, Nuuk, Greenland; 2 Department of Veterinary and Animal Sciences, University of Copenhagen, Copenhagen, Denmark; 3 Department of Internal Medicine, Queen Ingrid's Hospital Nuuk, Nuuk, Greenland; 4 Department of Infectious Disease Epidemiology & Prevention, Statens Serum Institut, Copenhagen, Denmark; 5 National Board of Health, Nuuk, Greenland; 6 Department of Epidemiology Research, Statens Serum Institut, Copenhagen, Denmark; 7 Department of Clinical Microbiology, Rigshospitalet University Hospital, Copenhagen, Denmark; 8 Department of Clinical Biochemistry, Bispebjerg University Hospital, Copenhagen, Denmark; 9 Clinical medicine / Public health, University of Aarhus, Aarhus, Denmark; 10 Ilulissat Regional Hospital, Ilulissat, Greenland; 11 Department of Infectious Diseases, Rigshospitalet University Hospital, Copenhagen, Denmark

**Keywords:** Long COVID, Greenland, Arctic, SARS-CoV-2, COVID-19

## Abstract

**Objectives:**

This study aimed to explore how the Greenlandic population experienced the course of both acute and long-term COVID-19. It was motivated by the unique epidemiologic situation in Greenland, with delayed community transmission of SARS-CoV-2 relative to the rest of the world.

**Methods:**

In a survey among 310 Greenlandic adults, we assessed the association between previous SARS-CoV-2 infection and overall health outcomes by administering three repeated questionnaires over 12 months after infection, with a response rate of 41% at the 12-month follow-up. The study included 128 individuals with confirmed SARS-CoV-2 infection from January/February 2022 and 182 test-negative controls. Participants were recruited through personal approaches, phone calls, and social media platforms.

**Results:**

A total of 53.7% of 162 participants who were test-positive recovered within 4 weeks and 2.5% were hospitalized due to SARS-CoV-2. The most common symptoms were fatigue and signs of mild upper respiratory tract infection. Less than 5% reported sick leave above 2 weeks after infection. Compared with participants who were test-negative, there was an increased risk of reporting fatigue (risk differences 25.4%, 95% confidence interval 8.8-44.0) and mental exhaustion (risk differences 23.4%, 95% confidence interval 4.8-42.2) up to 12 months after a positive test.

**Conclusions:**

Our results indicate that during a period dominated by the Omicron variant, Greenlanders experienced a mild acute course of COVID-19, with quick recovery, minimizing the impact on sick leave. Long COVID may be present in Greenlanders, with symptoms persisting up to 12 months after infection. However, it is important to consider the small sample size and modest response rate as limitations when interpreting the results.

## Highlights

• This study evaluated the acute and long-term effects of COVID-19 in Greenland.
• Greenland experienced a late and mild onset of COVID-19 community transmission.
• Over half made a full recovery within 4 weeks after a positive SARS-CoV-2 test.
• Fatigue was the most reported acute symptom.
• There is a higher risk of fatigue and mental exhaustion up to 12 months after SARS-CoV-2 infection.

## Introduction

Most individuals infected with SARS-CoV-2 recover relatively quickly after infection. However, some patients may experience short- or long-term late-onset complications; an overall risk of COVID-19 long-term complications is estimated at 10-30% among non-hospitalized cases and 50-70% among hospitalized cases [1]. Long-term complications after COVID-19 infection, also known as long COVID, have been defined by The World Health Organization (WHO) as the presence of persistent or new symptoms 3 months after a positive COVID-19 test. These symptoms must continue for at least 2 months and cannot be attributed to an alternative diagnosis [2]. Long COVID symptoms vary greatly, with WHO having identified over 200 symptoms [2]. The proportion and severity of long COVID symptoms also depend on the variant of concern with milder symptoms reported during periods dominated by the Omicron variant [3].

Although we find ongoing research on this topic elsewhere, long COVID has yet to be studied in an Arctic setting, such as Greenland. Greenland had a notably milder experience with the COVID-19 pandemic than other countries, which motivates a study of long COVID in this setting. In Greenland, community transmission did not occur until winter 2021 when the Omicron variant was predominant [4]. By June 2022, there had been a surge in COVID-19 cases; however, none of them had required ventilator support, and only 12 COVID-19–related deaths (21 deaths per 100,000 inhabitants) were reported [4]. The course of the epidemic in Greenland can likely be attributed to the early and strict public health and social measures and travel restrictions enforced by Greenlandic authorities, which delayed the introduction of COVID-19 to a period dominated by milder variants, such as Omicron [4,5]. Furthermore, this delay provided the national healthcare system time to vaccinate 67% of the population, reducing the risks of severe disease and death [4]. This was particularly crucial in Greenland, with a population potentially at an increased risk of severe COVID-19 disease. This is due to a high prevalence of comorbidities in the population, crowded living conditions, limited access to health care services, a well-documented high burden of infectious diseases, and historical accounts of the devastating consequences of past epidemics in circumpolar populations [6,7].

This unique situation raises the question of how the Greenlandic population experienced the acute- and long-term effects of COVID-19. Therefore, we aimed to investigate the frequency and severity of acute- and long-term COVID-19 symptoms in Greenland during a period dominated by the Omicron variant and compare these findings with other populations.

## Methods and materials

### Study design and population

The study was a cohort study and included adults aged above 18 years, living in Nuuk, with a self-reported positive polymerase chain reaction (PCR) or rapid antigen test for SARS-CoV-2 from January/February 2022. We also included a reference group who only had tested negative at the time of inclusion, matched 3:2. The oversampling of the reference group was due to the risk of non-infected individuals becoming infected later and thereby changing their exposure status. Participants in the test-negative group were transferred to the test-positive group if and when they had a positive test.

### Testing facilities in Greenland

In Greenland, access to PCR tests for SARS-CoV-2 was not available until April 2020. Before this, all PCR tests had to be sent to Denmark by flight for processing because Denmark had the necessary laboratory capacity. In July 2020, testing capacity expanded beyond Nuuk (the capital city) to include all four regional hospitals. PCR tests were provided free of charge and could be arranged through the Greenlandic health care system. Initially, PCR tests were accessible to all individuals in Greenland, regardless of symptoms. However, by the winter of 2021/2022, the surge in case numbers overwhelmed the country's testing facilities, forcing the Greenlandic health authorities to restrict PCR tests to individuals at a higher risk of severe disease. Those experiencing COVID-19 symptoms were advised to self-isolate or use a rapid antigen test instead. Throughout much of the pandemic, the population was encouraged to use rapid antigen tests due to the limited availability of PCR testing. These rapid tests became available in mid-2020 at healthcare clinics and could also be purchased in stores. The results of rapid antigen tests were not reported to the Greenlandic health authorities [4].

### Recruitment methods

We recruited participants using different approaches. First, we obtained SARS-CoV-2 PCR test results from the Greenlandic electronic medical records system conducted in January/February 2022. Test results and dates, along with contact information for the tested individuals, were given by the Greenlandic health authorities. The identified individuals (test-positive and test-negative) were then contacted by telephone and invited to participate. Second, we recruited participants who had tested positive by PCR test or rapid antigen test in the same time period, as well as those who had tested negative, using social media platforms and personal approaches in either Greenlandic or Danish, including engagement at the local shopping mall. Due to logistic limitations, participants were only recruited in Nuuk. It was not possible to record the number of individuals invited to complete the questionnaires.

To reduce recall bias, we restricted our analysis to participants who had undergone testing within, approximately, the past 3 months. In addition, we ensured that all participants, regardless of their test result, were asked the same set of questions to reduce potential reporting bias.

### Data

Outcome data were collected through three questionnaires received at approximately 3 (baseline), 6, and 12 months after a positive test for SARS-CoV-2 or the inclusion date for participants who were test-negative. The first questionnaire was launched on May 23, 2022, when the Omicron variant dominated in Greenland [4]. The data collection schedule was based on the WHO definition of long COVID, which is defined as the continuation or development of new symptoms 3 months after the initial SARS-CoV-2 infection [8].

The questionnaires were available in Greenlandic and Danish and adapted to Greenlandic circumstances. Data were created and collected using Microsoft Forms, an online survey platform.

The first questionnaire was completed in person, allowing participants to seek clarification or additional explanations from study representatives if they had any uncertainties regarding the questions. The second and third questionnaires were mailed to participants and completed online.

The questionnaires consisted of questions regarding height, weight, education, employment, place of birth, smoking and drinking habits, physical activity, sick leave, self-reported health, preexisting health conditions, and contact information. Participants who had tested positive also provided information regarding the symptoms they experienced during their initial infection, the severity of acute disease, and their current recovery status. The severity of acute disease was categorized into three levels of declining severity: hospitalization, mild to moderate symptoms but did not require hospitalization, and asymptomatic. To evaluate the post-acute COVID-19 symptoms, all participants were asked about specific physical and psychological symptoms, cognitive challenges, and fatigue experienced in the past 2 weeks, as well as any preselected health conditions diagnosed since the last completed questionnaire.

Data regarding COVID-19 vaccination status among the participants were not collected. This was attributed to the fact that not all vaccinations were recorded in the Greenlandic health registries. Some participants could have received vaccine doses in other countries, such as Denmark, which were not documented in Greenland. Therefore, we chose not to include vaccination data in this study due to the potential for missing or incomplete information.

All participants were asked about their sick leave history in the period since the previous completed questionnaire. Those who confirmed having taken sick leave were subsequently questioned about the duration of their leave. Sick leave lasting more than 2 weeks was considered significant.

For individuals who had never tested positive for SARS-CoV-2, we provided the option to analyze their blood samples for anti-nucleoprotein immunoglobin (Ig) total antibodies [9]. This analysis aimed to determine whether they had previously been infected with SARS-CoV-2 despite negative test results. The purpose of this analysis was to estimate the overall positivity rate within the presumed test-negative group and avoid misclassification bias. If a participant had a positive level of total Ig anti-nucleoprotein (N-positivity), they were transferred to the test-positive group.

The questionnaires and methodology of this study were partly inspired by the Danish long COVID study “EFTER-COVID” [10]. The questionnaire is available as supplementary material (Supplementary Note 1).

### Blood sampling and laboratory test

Blood samples were sent to Denmark, where the antibody analyses were performed at the Department of Clinical Biochemistry at Bispebjerg University Hospital, Denmark.

The subgroup of participants who were test-negative had a venous blood sample taken using an ethylenediamine tetraacetic acid tube. Blood samples were centrifuged (4000 g for 10 minutes), serum collected, frozen within 4 hours from collection, and kept at −20°C during transportation to the laboratory in Denmark.

The total (IgG+A+M) SARS-CoV-2 nucleoprotein antibody (N-Ab) levels were measured using ECLIA Assays (Roche Diagnostics, Mannheim, Germany) [9]. N-positivity was defined as levels exceeding 0.8 cut-off index [11].

### Statistical analysis

We used the Kruskal–Wallis test for continuous variables and Pearson's chi-square test/Fisher's exact test for categorical variables. Medians and interquartile range (IQR), defined as the range from the 25th percentile to the 75th percentile of the data when sorted in ascending order, were calculated for continuous variables.

The Charlson comorbidity index (CCI) scores were calculated based on self-reported health data. The score assigns a weight (1, 2, 3, or 6) to each of the 19 major disease categories and is a validated measure of comorbidity [10]. We categorized the scores as 0, 1, and 2 or more. Although the CCI is constructed for register-based studies, we categorized comorbidity based on questionnaire information using the CCI disease groupings.

We compared the prevalence of conditions and symptoms between individuals who tested positive and those who had tested negative for SARS-CoV-2 using risk differences (RDs). We calculated the RDs for the following conditions: (i) selected post-acute physical symptoms experienced in the time around the completion of the questionnaire at 6 or 12 months after the test or inclusion, (ii) new medical diagnoses confirmed by a medical doctor since the test or inclusion (with onset between the time of testing and questionnaire completion), and (iii) new occurrences of various health problems since the test or inclusion (with onset between the time of testing and questionnaire completion).

RDs were estimated using parametric g-computation and logistic regression analysis. We adjusted for variables relevant to the study of long COVID, inspired by other studies [10]: age, gender, body mass index, comorbidities, and Inuit ethnicity. We based each bootstrap confidence interval (CI) on 1000 resamples. RDs and 95% CIs are shown in percentages.

*P*-values ≤0.05 were considered statistically significant.

Data analysis and visualization were conducted using R, version 4.2.3 [12] and the R-package “riskCommunicator” to calculate the RDs and CIs [13]. For visualization and creation of forest plots, we used the “forestplot” R-package.

## Results

The study included a total of 310 participants, where 41% (n = 128) had tested positive for SARS-CoV-2 and 59% (n = 182) had never tested positive. In the test-positive group, 59% were females and 86% were born in Greenland. The median age was 46 years. In the test-negative group, 64% were female, 75% were born in Greenland, and the median age was 51 years (Table 1).

**Table 1:** Demographics of 310 participants by test result for SARS-CoV-2.

N total = 310	Test positive N = 128	Test negative N = 182	*P*-value
N Person (%)			0.379
Male	52 (40.6)	65 (35.7)	
Female	76 (59.4)	117 (64.3)	

Age at enrolment (years)			0.317
Median (IQR)	46 (36-57)	51 (38-60)	

N Birth place (%)			0.060
Greenland	110 (85.9)	137 (75.3)	
Denmark	17 (13.3)	41 (22.5)	
Other	1 (0.8)	4 (2.2)	

N Education level (%)			0.150
Primary or elementary school (equivalent to 9th-10th grade)	12 (9.4)	21 (11.5)	
General secondary or vocational secondary	9 (7.0)	13 (10.2)	
Vocational training	26 (20.3)	22 (12.1)	
Higher (1-2 years, vocational academy)	15 (11.7)	12 (6.6)	
Higher education (2-4 years, BSc)	43 (33.6)	84 (46.2)	
Higher (>5 years, MSc, PhD)	21 (16.4)	24 (13.2)	
Other	2 (1.6)	4 (2.2)	

N Employment (%)			0.782
Part-time employed	2 (1.6)	2 (1.1)	
Full-time employed	95 (74.2)	128 (70.3)	
Stay-at-home or on parental leave	2 (1.6)	6 (3.3)	
Jobseeker/Unemployed	2 (1.6)	3 (1.6)	
Long-term sick leave	1 (0.8)	0 (0)	
Pensioner or early retiree	6 (4.7)	15 (8.3)	
Self-employed	8 (6.3)	9 (4.9)	
Student	9 (7.0)	12 (6.6)	
Other	3 (2.3)	7 (3.8)	

N Charlson comorbidity score index (%)			0.863
0	112 (87.5)	161 (88.5)	
1	14 (10.9)	19 (10.4)	
2 or more	2 (1.6)	2 (1.1)	

N Smoking status (%)			0.772
Former smoker	53 (41.4)	68 (37.4)	
Current smoker	42 (32.8)	64 (35.2)	
Never smoked	33 (25.8)	50 (27.5)	

N Reported alcohol consumption (%)			0.352
Yes	71 (55.5)	92 (50.5)	
No	54 (42.2)	87 (47.8)	

Body mass index			0.317

Median (IQR)	28.0 (25.1-31.6)	29.3 (25.3-32.2)	

N Self-reported assessment of health (%)			0.168
Poor	29 (22.7)	30 (16.5)	
Good	54 (42.2)	70 (38.5)	
Very good	45 (35.2)	82 (45.5)	

N Self-reported physical activity (%)			0.754
Reads, watch TV, or other sedentary lifestyle	37 (28.9)	60 (33.0)	
Work out or do gardening (at least 4 hours/week)	22 (17.2)	26 (14.3)	
Walks, cycles, or light exercise (at least 4 hours/week)	62 (48.4)	87 (47.8)	
Hard training or competitive sports (several times/week)	6 (4.7)	6 (3.3)	

*P*-values were estimated using the Kruskal–Wallis test for continuous variables and Pearson's chi-square test/Fisher's exact test for categorical variables.

Of 142 (78%) participants in the test-negative group who provided a blood sample, 24% (n=34) exhibited N-positivity.

In total, 179 (58%) individuals completed a follow-up questionnaire at 6 months and 128 (41%) at 12 months (Supplementary Figure 1).

### Participants who were test-positive for SARS-CoV-2

#### Acute symptoms from 1 week before until 4 weeks after test

Of the 162 participants who tested positive for SARS-CoV-2, 2.5% required hospitalization, whereas 74.6% experienced mild to moderate symptoms but did not require hospitalization and 22.9% were asymptomatic.

Specifically, 75% reported at least one acute symptom in the interval from 1 week before the test until 4 weeks after the test, with a median of four symptoms (IQR 1-9 symptoms). The greater severity of acute SARS-CoV-2 infection was associated with a higher number of reported symptoms, indicating that hospitalized individuals experienced a greater number of acute symptoms (*P*-value <0.001). The most common acute symptoms of SARS-CoV-2 infection were fatigue (72%), headache (68%), stuffy/runny nose (57%), cough (56%), and muscle/joint pain (55%) (Supplementary Figure 2).

A total of 4 weeks after a positive test, individuals self-rated their health as follows: 54% reported a return to pre-illness levels, 29% indicated that their health had not fully recovered, and 17% expressed uncertainty regarding their health status.

Based on descriptive results, women aged 30-39 years tended to report acute and post-acute symptoms more often. Inuit ethnicity, education, occupation, lifestyle factors such as smoking/alcohol habits, body mass index, physical activity level, and self-reported health were not associated with a higher reported number of either acute or post-acute COVID-19 symptoms.

#### Post-acute symptoms, health problems, and new medical diagnoses

At the 6-month follow-up, 71% (91 of 128 individuals who were test-positive) of all the included participants who were test-positive reported experiencing at least one long COVID symptom, which declined to 55% (70 of 128 individuals who were test-positive) at the 12-month follow-up.

Fatigue was the most frequently reported symptom among those who tested positive, with a prevalence of 46.5% at 6 months and 40.4% at 12 months after testing. Muscle/joint pain (37.8% and 33.3%) and dyspnea (22.0% and 21.2%) were also frequently reported symptoms at both time points (Table 2).

**Table 2:** Point prevalence of physical and mental symptoms, health problems, and new medical diagnosis among participants who were test-positive and test-negative at 3, 6, and 12 months after a SARS-CoV-2 test or the inclusion date.

	Test-positive participants			Test-negative participants		
	3 months (baseline) N total 162 prevalence (%)	6 months N total 127 prevalence (%)	12 months N total 99 prevalence (%)	3 months (baseline) N total 148 prevalence (%)	6 months N total 52 prevalence (%)	12 months N total 28 prevalence (%)
**Physical symptoms**						
*Smell distortion*	37 (22.8)	16 (12.6)	21 (21.2)	-	3 (5.8)	2 (7.1)
*Taste distortion*	33 (20.4)	14 (11.0)	17 (17.2)	-	3 (5.8)	0 (0)
*Dyspnea*	64 (39.5)	28 (22.0)	21 (21.2)	-	9 (17.3)	7 (25.0)
*Chest pain*	18 (11.1)	17 (13.4)	10 (10.1)	-	7 (13.5)	3 (10.7)
*Muscle joint pain*	64 (39.5)	48 (37.8)	33 (33.3)	-	14 (26.9)	7 (25.0)
*Fatigue*	86 (53.1)	59 (46.5)	40 (40.4)	-	17 (32.7)	5 (17.9)
*Fever/chills*	64 (39.5)	24 (18.9)	14 (14.1)	-	5 (9.6)	4 (14.3)
**Mental symptoms**						
*Anxiety symptoms*	7 (4.3)	19 (15.0)	15 (15.2)	8 (5.4)	6 (11.5)	5 (17.9)
*Depressive symptoms*	3 (1.9)	6 (4.7)	1 (1.0)	10 (6.8)	2 (3.8)	0 (0)
**Health problems**						
*Physical exhaustion*	-	58 (46.8)	38 (40.4)	-	14 (26.9)	8 (28.6)
*Mental exhaustion*	-	56 (45.5)	41 (44.1)	-	11 (21.2)	5 (17.9)
*Difficulties concentrating*	-	40 (32.5)	28 (32.9)	-	8 (15.4)	6 (21.4)
*Memory issues*	-	40 (33.6)	30 (33.7)	-	5 (9.6)	7 (25.0)
*Sleep problems*	-	50 (41.3)	33 (36.7)	-	19 (36.5)	6 (21.4)
**Medical diagnosis**						
*Hypertension*	-	12 (9.4)	8 (8.0)	-	1 (1.9)	2 (7.1)
*Chronic fatigue syndrome*	-	3 (2.4)	3 (3.0)	-	0 (0)	0 (0)
*Diabetes*	-	3 (2.4)	2 (2.0)	-	0 (0)	0 (0)
*PTSD (Post-traumatic stress disorder)*	-	1 (0.8)	1 (1.0)	-	0 (0)	0 (0)
*Fibromyalgia*	-	1 (0.8)	1 (1.0)	-	0 (0)	0 (0)
*Depression diagnosis*	-	4 (3.1)	2 (2.0)	-	2 (3.8)	0 (0)
*Anxiety diagnosis*	-	6 (4.7)	2 (2.0)	-	4 (7.7)	2 (7.1)
*Chronic obstructive pulmonary disease /chronic lung disease*	-	1 (0.8)	1 (1.0)	-	1 (1.9)	1 (3.6)
*Asthma*	-	5 (3.9)	2 (2.0)	-	4 (7.7)	2 (7.1)
*Chronic headache/migraine*	-	6 (4.7)	3 (3.0)	-	1 (1.9)	0 (0)

Physical and mental exhaustion were the most frequent new health problems reported throughout the 12-month follow-up period. However, it is worth noting that the prevalence of these symptoms showed a decreasing trend over time (Table 2).

Hypertension was the only new medical diagnosis given by a medical doctor since the last questionnaire that was relatively often reported, with a prevalence of 9.4% at 6 months and 8% at 12 months after testing. All other medical diagnoses had a prevalence of less than 5% during the study period (Table 2).

### Comparison of participants who were test-positive and participants who were test-negative for SARS-CoV-2

#### Post-acute symptoms

**Figure 1:**
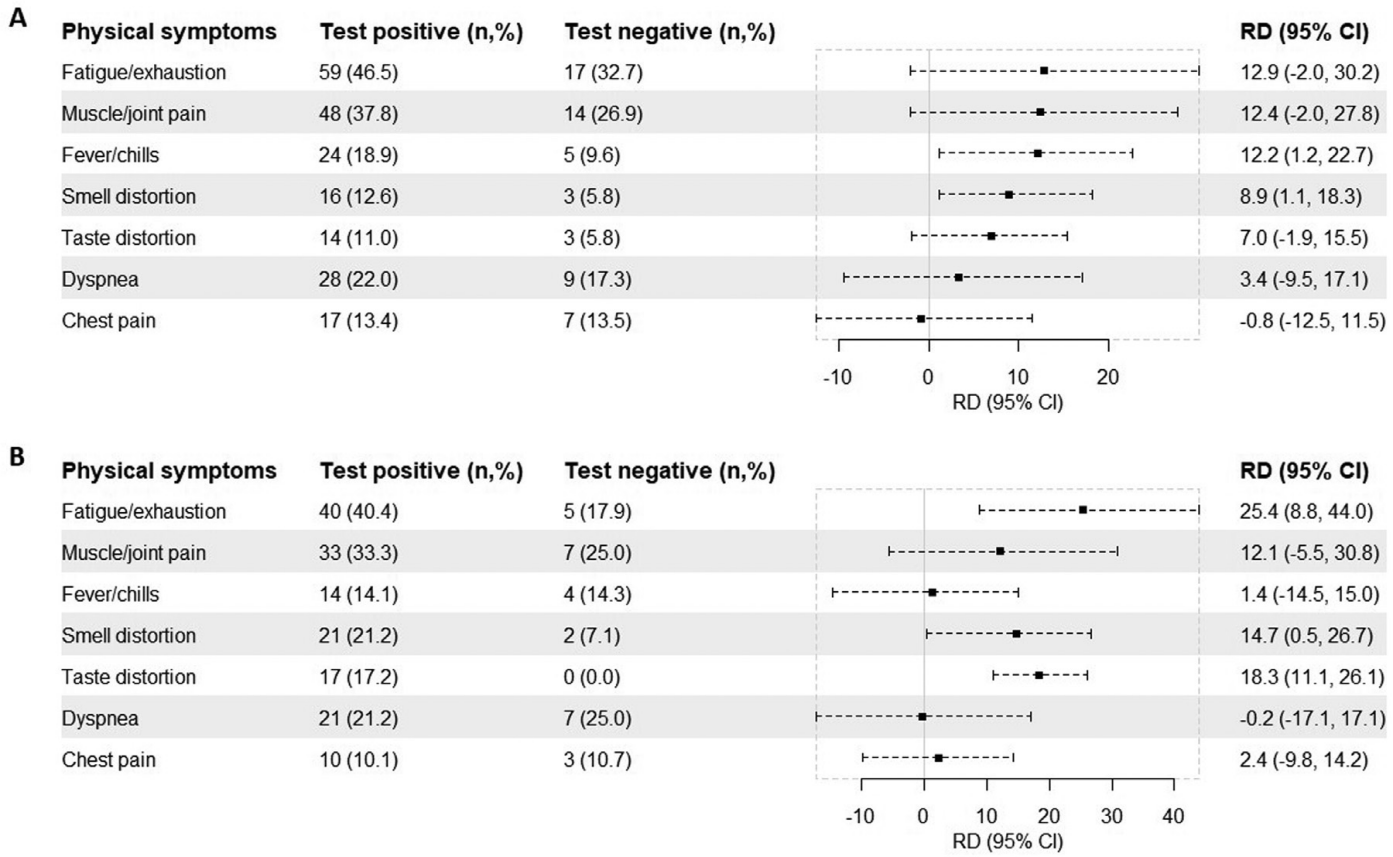
Risk differences of physical symptoms at 6 (A) and 12 (B) months after test date or inclusion date, comparing participants who were test-positive and test-negative for SARS-CoV-2. Bars indicate risk differences (center), with 95 confidence intervals (length of error bars). Adjusted for age, gender, body mass index, comorbidities, and Inuit ethnicity. All proportions are based on 127 participants who were test-positive and 52 participants who were test-negative at 6 months (A) and 99 participants who were test-positive and 28 participants who were test-negative at 12 months (B) after test or inclusion date. CI, confidence interval; RD, risk differences.

The most noteworthy differences in risk (RDs) at the 6-month follow-up were observed concerning fatigue/exhaustion (RD 12.9%, 95% CI −2.0-30.2), muscle/joint pain (RD 12.4%, 95% CI −2.0-27.8), and fever/chills (RD 12.2%, 95% CI 1.2-22.7). At the 12-month follow-up, we found a higher statistical significance in relation to fatigue/exhaustion (RD 25.4%, 95% CI 8.8-44.0), taste distortion (RD 18.3%, 95% CI 11.1-26.1), and smell distortion (RD 14.7%, 95% CI 0.5-26.7) (Figure 1).

#### New medical diagnoses and health problems

**Figure 2:**
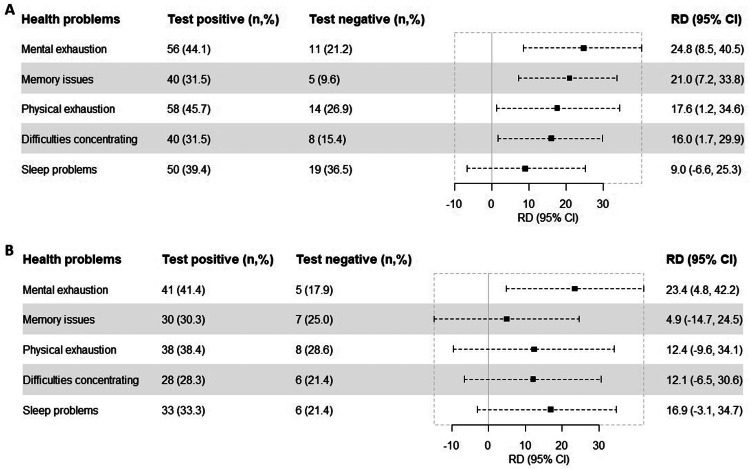
Risk differences of self-reported new onset health problems at 6 (A) and 12 (B) months after test date or inclusion date, comparing participants who were test-positive and test-negative for SARS-CoV-2. Bars indicate risk differences (center), with 95 confidence intervals (length of error bars). Adjusted for age, gender, body mass index, comorbidities, and Inuit ethnicity. All proportions are based on 127 participants who were test-positive and 52 participants who were test-negative at 6 months (A) and 99 participants who were test-positive and 28 participants who were test-negative at 12 months (B) after test or inclusion date. CI, confidence interval; RD, risk differences.

All health problems tended to be more common among participants who were test-positive than participants who were test-negative. The health problems with the largest RDs at 6 and 12 months were mental exhaustion (RD 24.8%, 95% CI 8.5-40.5 and RD 23.4%, 95% CI 4.8-42.2) and physical exhaustion (RD 17.6%, 95% CI 1.2-34.6 and RD 12.4%, 95% CI −9.6-34.1), respectively (Figure 2).

**Figure 3:**
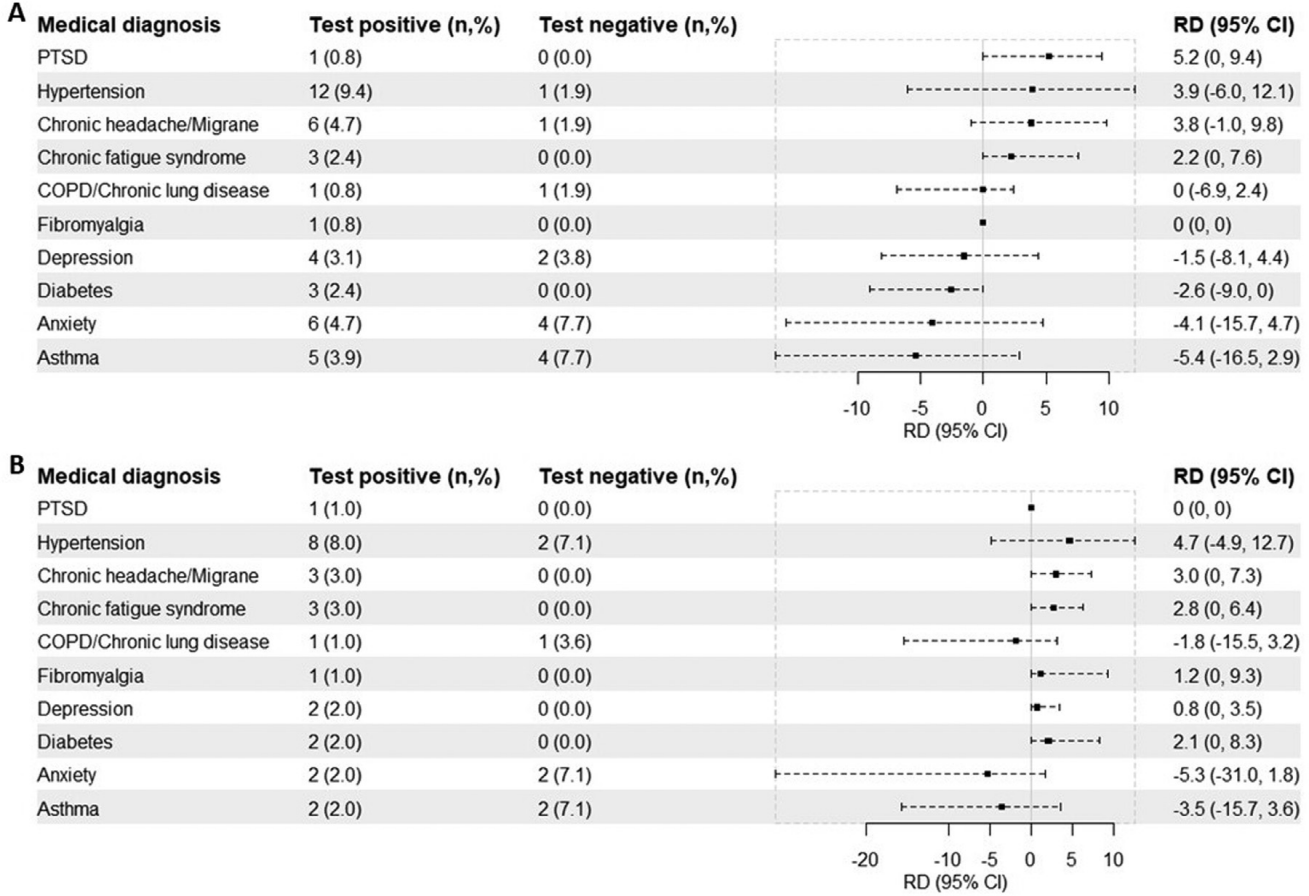
Risk differences of self-reported new medical diagnoses at 6 (A) and 12 (B) months after test date or inclusion date, comparing participants who were test-positive and test-negative for SARS-CoV-2. Bars indicate risk differences (center), with 95 confidence intervals (length of error bars). Adjusted for age, gender, body mass index, comorbidities, and Inuit ethnicity. All proportions are based on 127 participants who were test-positive and 52 participants who were test-negative at 6 months (A) and 99 participants who were test-positive and 28 participants who were test-negative at 12 months (B) after test or inclusion date. CI, confidence interval; RD, risk differences; PTSD, Post-traumatic stress disorder.

We found non-significant RDs between the test groups regarding the included new medical diagnosis. The largest RDs were found for chronic headache (RD 3.8%, 95% CI −1.0-9.8 and RD 3.0%, 95% CI 0-7.3), chronic fatigue syndrome (RD 2.2%, 95% CI 0-7.6 and RD 2.8%, 95% CI 0-6.4), and hypertension (RD 3.9%, 95% CI −6.0-12.1 and RD 4.7%, 95% CI −4.9-12.7) at both time points (Figure 3).

#### Sick leave

The prevalence of sick leave lasting more than 2 weeks in individuals who tested positive was 5.0% (95% CI 1.45-8.55%) at 6 months and 3.9% (95% CI 0.10-7.69%) at 12 months. In those who tested negative, it was 1.1% (95% CI 0.22-1.98%) at 6 months and 0.8% (95% CI 0.04-1.56%) at 12 months. The prevalence remained similar across age groups and gender, regardless of test status (Supplementary Figures 3 and 4).

## Discussion

### Main findings

We found that the majority of individuals who tested positive for SARS-CoV-2 experienced a relatively mild and short acute course of the disease, which aligns with findings from other studies evaluating infection severity during the Omicron period [3,14]. Given that over half of the participants had fully recovered within the first month after testing positive, our results indicate that most infected individuals did not report any long-term effects from the infection. This aligns with findings from other studies, which observed a lower prevalence and risk of reporting long COVID symptoms after Omicron infection than infection with pre-Omicron variants [15], [16], [17]. An important finding was that the prevalence of sick leave remained consistently below 5% throughout the study period, irrespective of test status, which is equivalent to findings in a Danish study [18]. This suggests that the reported post-acute symptoms, regardless of their severity, did not affect work attendance to a large degree. Moreover, the declining prevalence of sick leave throughout the study period suggests a potential reduction in the severity and duration of long COVID symptoms.

Fatigue was the predominant symptom reported among individuals who were test-positive throughout the study, followed by mental and physical exhaustion. Cognitive issues, including memory and concentration problems, as well as sleep disturbances, were also prevalent, with over a third of individuals who were test-positive reporting them up to 12 months after testing. These findings align with observations in other populations [1,10,14,19,20]. When comparing the risk of reporting long COVID symptoms between the test-positive and test-negative groups, we consistently found moderate to low RDs, suggesting that the prevalence of long COVID symptoms in Greenland may be relatively low. However, given the small study population and the moderate response rate, it is important to consider these limitations when generalizing our results.

We did not observe a higher risk of developing new medical diagnoses after a positive test for SARS-CoV-2, unlike findings reported in other studies [10,21]. However, this observation could be attributed to the limited sample sizes available for analysis.

We found that women aged 30-39 years were more likely to report symptoms, which is consistent with findings from several previous studies that have identified female sex as a consistent risk factor [10,22,23]. However, the existing evidence on the influence of age on long COVID remains somewhat conflicting [10,23]. Furthermore, the results might have been affected by selection bias due to the predominantly young population in our study, which is reflective of Greenland's overall young demographic profile [24].

### Perspectives

Direct comparison with other populations is challenging due to various factors, including differences in methodology (e.g. studies lacking a negative control group), study population characteristics (e.g. sample size, and inclusion of only hospitalized participants), the timing of the COVID-19 pandemic (dominant variant type), and the type and number of symptoms assessed. Many studies have shown a high prevalence of long COVID in several populations, including a comprehensive Danish study that included a test-negative control group for comparison [10,19,22,25,26]. However, other studies using a methodology similar to ours found no difference in the prevalence of symptoms potentially associated with long COVID in the case and control groups [27,28].

As mentioned, Greenland, in theory, appeared to be at a higher risk of experiencing severe consequences of the COVID-19 pandemic despite having a relatively young population. However, successful early implementation of a containment strategy delayed the virus and allowed time for vaccination. This, along with the dominance of milder variants such as Omicron, reduced the impact of the pandemic. The combined strategies used by the Greenlandic health authorities minimized the risk of severe coronavirus disease and may have influenced the occurrence of long COVID through a high vaccination rate [4], which previous studies have shown to provide protection against long COVID [1,14], infection with milder variants, and a mild clinical course of infection.

Genetic factors may also have played a role in the course of COVID-19 infection in Greenland, despite our study not finding any difference in long COVID occurrence based on ethnicity, unlike other studies [20,29,30]. Although genetic defects linked to COVID-19 severity have been identified, none have been linked to the risk of developing long COVID [31]. However, future research will likely discover a limited number of genetic variants that contribute to the pathology, similar to recent findings of the IFNAR2 gene defect in the Inuit population, which causes defects in the individuals’ antiviral defense mechanism [32].

### Strengths and limitations

Our study has several strengths. First, it incorporated a test-negative control group, offering valuable insights into the prevalence of symptoms associated with long COVID within the broader Greenlandic population. In addition, the inclusion of serological evidence (N-positivity) for previous SARS-CoV-2 infection in the test-negative group helped mitigate potential misclassification bias. Finally, our questionnaires were available in Danish and Greenlandic, which may have improved participation and the validity of the collected data.

It is important to address the study's limitations, notably, the relatively small sample size, with only 41% of participants completing all three questionnaires, possibly causing selection bias. This could affect the generalizability of our findings to the broader population of Greenland and other populations. It could also have led to a small-study effect, potentially leading to overestimation of symptom prevalence. In addition, by recruiting solely from the capital city of Greenland, our sample may not fully represent the diversity of the entire population. Demographics, cultural factors, and living habits in the capital city could differ significantly from those in more remote regions. Furthermore, our recruitment methods, including personal approaches in public spaces and social media platforms, may introduce bias and overestimation of symptom burden by attracting participants who were more likely to volunteer, potentially skewing the results toward individuals who experienced COVID-19 symptoms and were more willing to participate. Furthermore, the risk of information bias is to be considered because participants who are aware of having had a COVID-19 infection can be more likely to report the presence of ongoing symptoms [33].

We chose not to exclude a small number of test-negative participants who tested positive for SARS-CoV-2 during or at the beginning of the study, despite not knowing the time of infection. Instead, they were categorized as test-positive individuals. This approach could have introduced uncertainty regarding estimating the proportion of long COVID symptoms at the specified follow-up time points.

Our study focused on a limited set of the over 200 registered long COVID symptoms, potentially missing the frequency of other symptoms that could indicate long COVID [2]. Furthermore, cultural differences may impact the likelihood of reporting long COVID symptoms [34]. It is important to consider that the questionnaire used in our study was inspired by the Danish long COVID study, which poses potential challenges due to the social and cultural differences between Greenland and Denmark. These differences can lead to misunderstandings and varying interpretations of the questions asked and thus potentially confound the results. Similar challenges have been reported in previous research conducted within Arctic communities [6].

## Conclusion

In conclusion, our findings show a relatively mild acute disease of COVID-19 among Greenlanders during the Omicron-dominated period. Although our data do not suggest a high burden of long COVID symptoms within the 12 months after infection among Greenlanders, it may remain a notable health concern for those previously infected.

## Declarations of competing interest

The authors have no competing interests to declare.

## Appendix Supplementary materials

Image, application 1
