# Supplementary material for: Exploring the dynamics of COVID-19 in a Greenlandic cohort: Mild acute illness and moderate risk of long COVID
Source: IJID Reg. 2024 Apr 14;11:100366. doi: 10.1016/j.ijregi.2024.100366 (PMC11081797; doi:10.1016/j.ijregi.2024.100366)
Supplement: Supplementary file 1 [file mmc1.docx]

**Supplementary Material**

**Supplementary Figure 1** Flowchart illustrating participant enrollment at 3, 6, and 12 months after the test or inclusion date, and the grouping distribution stratified by SARS-CoV-2 test status.


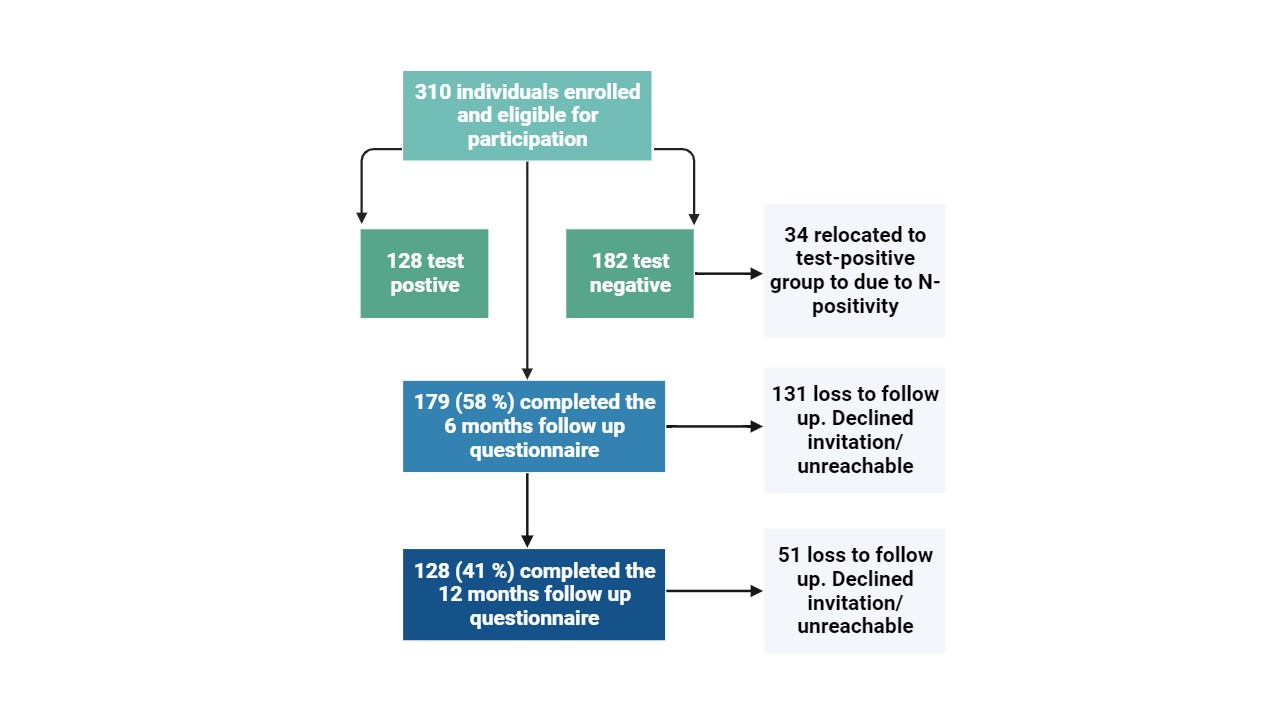


SARS-CoV-2 test-negative participants gave blood samples for testing of anti-nucleoprotein immunoglobin (Ig) total antibodies. N-positivity was defined as levels exceeding 0.8 COI (cut-off index).


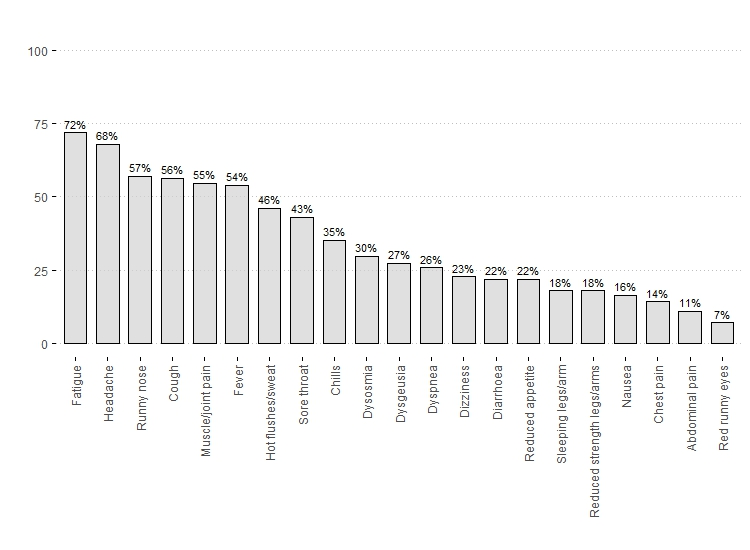
**Supplementary Figure 2** Prevalence (%) of acute symptoms in infected individuals observed from 1 week before to 4 weeks after a positive SARS-CoV-2 test.

**Supplementary Figure 3** Unadjusted prevalence (%) of sick leave, defined as >2 weeks of sick leave in the period since the last questionnaire at 6 months (**A**) and 12 months (**B**) after the test or inclusion date.

**Supplementary Figure 4** Unadjusted prevalence (%) of sick leave in the period since the last questionnaire at 6 (**A**) and 12 (**B**) months after the test date, stratified by age, comparing SARS-CoV-2 test-positive and test-negative participants.

**
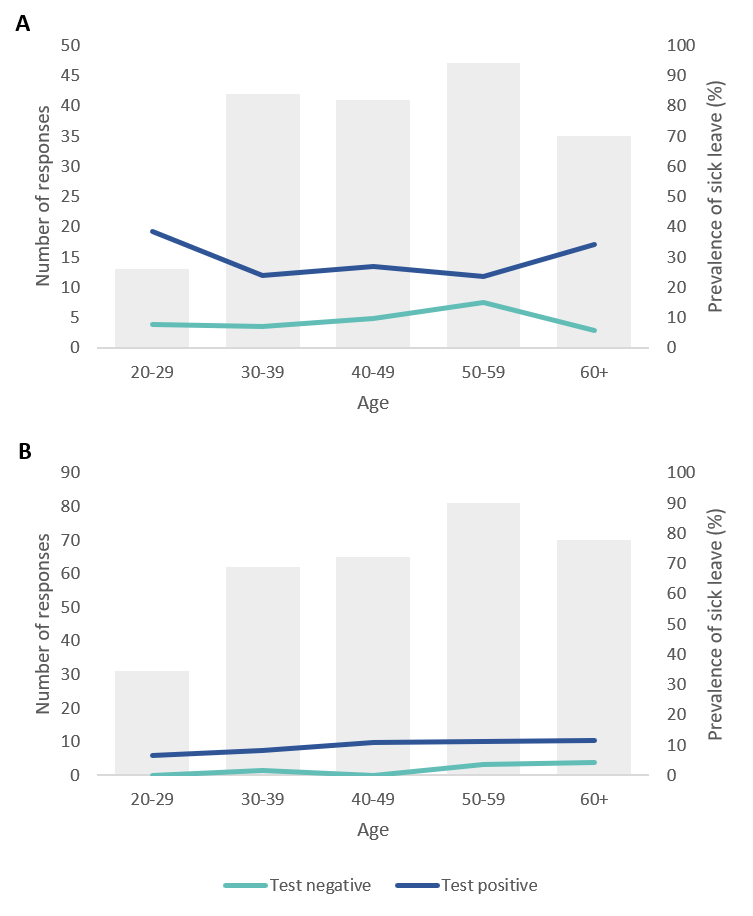
**

**Supplementary Note 1** English translation of questionnaires

**Questionnaire – baseline (3 months)**

*(Introductory message to participants)*

Welcome to the Greenlandic study on COVID-19 long-term effects.

In this study, we investigate the population's health during and after the coronavirus pandemic. Your responses are important, whether you have tested positive or negative for coronavirus (COVID-19).

Everyone invited to participate in the study is randomly selected to receive a questionnaire with a special focus on their health. The questionnaire specifically addresses physical symptoms, mental well-being, concentration, memory, as well as fatigue, both physically and mentally.

Thank you very much for your assistance and participation.

(*All* *participants were asked to complete the following section)*

Have you ever tested positive for COVID-19?

1) Yes, by a PCR-test

2) Yes, by a rapid antigen test / self-test

3) No, I have never had a positive test

Please provide your full name

____________________________

Please provide your CPR number (personal Danish ID number)

____________________________

Please provide your age

____________________________

**Background questions about health, education, work, and lifestyle.**

Please provide your approximate height in cm.

____________________________

If you don't know your height, simply proceed to the next question.

Please provide your approximate weight in kg.

____________________________

If you don't know your weight or prefer not to answer, please move on to the next question.

In which country were you born?

(1) Greenland

(2) Denmark

(3) Other

What is your highest completed education?

(1) Primary school / lower secondary school (equivalent to 9th-10th grade)

(2) General upper secondary education or vocational upper secondary education, e.g., business school and technical college

(3) Vocational education, e.g., craft apprenticeship

(4) Short-cycle higher education (1-2 years), e.g., health assistant

(5) Medium-cycle higher education (2-4 years), e.g., nurse, primary school teacher

(6) Long-cycle higher education (>=5 years), e.g., master's or Ph.D.

(7) Don't know / None of the above / Prefer not to answer

What is your main occupation?

(1) Full-time employed

(2) Part-time employed

(3) Self-employed

(4) Student

(5) Stay-at-home parent or on parental leave

(6) Job seeker / Unemployed

(7) Welfare recipient

(8) Long-term sick leave

(9) Retired or on disability pension

(10) Other

Do you smoke or have you smoked in the past?

(1) I have never smoked

(2) It has been more than 5 years since I smoked

(3) I smoked within the last 5 years but no longer do

(4) I smoke occasionally, e.g., at social events

(5) I smoke daily (less than 10 cigarettes/cigars/pipes a day)

(6) I smoke daily (10 or more cigarettes/cigars/pipes a day)

Please indicate how many alcoholic drinks you consume on average per week.

One drink could be, for example, 1 beer (33 cl), 1 glass of wine (12 cl), or 1 glass of spirits (4 cl).

____________________________

Looking at the last six months before the test date, how would you describe your physical activity during leisure time?

(1) Trains hard and regularly participates in competitive sports several times a week

(2) Engages in recreational sports or performs heavy indoor/outdoor work or similar for at least 4 hours a week

(3) Walks, cycles, or engages in other light exercise for at least 4 hours a week (including Sunday walks, light indoor/outdoor work, and cycling/walking to work)

(4) Reads, watches television, or has other sedentary activities

How would you rate your overall health in the last 14 days?

(1) Excellent

(2) Very good

(3) Good

(4) Fair

(5) Poor

Do you have any chronic illnesses such as asthma, COPD, heart disease, or diabetes? If yes, please specify.

____________________________

*(Only test-positive participants were asked to complete the following section)*

First, there are some questions about how you have been since you tested positive.

Which of the following conditions best describes how you felt when you were at your worst?

Think about the period from one week before you tested positive until 4 weeks after.

(1) I had no symptoms

(2) I was sick at home

(3) I was hospitalized due to COVID-19

(4) I was hospitalized due to COVID-19 and received ventilator treatment

(5) I was hospitalized for reasons other than COVID-19

Have you been on sick leave from work/studies due to COVID-19?

(1) Yes, I was on sick leave due to symptoms

(2) Yes, I was on sick leave, but only to avoid spreading the virus to others

(3) No, I was not on sick leave, as I only had mild/no symptoms

(4) No, I was not on sick leave (for other reasons)

Did your health fully return to how it was before COVID-19 within 4 weeks after you tested positive? If you never had symptoms, write 0 days below.

(1) My health is still not as it was before I got sick

(2) My health returned to normal after the onset of illness symptoms (write the number of days in the field below) _______

(3) Don't know

In the week leading up to your test and in the subsequent four weeks, have you experienced any of the following symptoms?

Please mark all the symptoms you have had, regardless of the cause.

|  | **Mark X** |
| --- | --- |
| Fever |  |
| Chills |  |
| Hot flashes or sweats |  |
| Headache |  |
| Muscle or joint pain |  |
| Fatigue or exhaustion |  |
| Dizziness |  |
| Red or watery eyes |  |
| Runny or blocked nose |  |
| Decreased or altered sense of smell |  |
| Decreased or altered sense of taste |  |
| Sore throat or swallowing pain |  |
| Cough |  |
| Shortness of breath |  |
| Chest pain |  |
| Nausea or vomiting |  |
| Diarrhea |  |
| Abdominal pain |  |
| Decreased appetite or aversion to food |  |
| Decreased strength in legs and arms |  |
| Numbness or tingling sensation or other sensory disturbances in legs and arms |  |

The following questions are answered only if you have selected yes to the corresponding symptom in the previous section. If you haven't answered yes to the above symptoms, please proceed to the next section.

How severe was your headache when you were at your worst?

(1) No pain

(2) Mild pain

(3) Moderate pain

(4) Severe pain

(5) Unbearable pain

How severe were your muscle or joint pains when you were at your worst?

(1) No pain

(2) Mild pain

(3) Moderate pain

(4) Severe pain

(5) Unbearable pain

How was your sense of taste different from normal?

My sense of taste was...

(1) Slightly impaired

(2) Significantly impaired

(3) Completely lost

(4) Altered

(5) Don't know

How was your sense of smell different from normal?

My sense of smell was...

(1) Slightly impaired

(2) Significantly impaired

(3) Completely lost

(4) Altered

(5) Don't know

Select below the statement(s) that best describe your breathing difficulties.

(1) I only get breathless when I exert myself greatly

(2) I get breathless when I hurry or walk up a slight hill

(3) I walk slower than others my age because of my breathlessness, or I have to stop to catch my breath when walking on level ground

(4) I stop to catch my breath after about 100 meters or a few minutes of walking

(5) I am too breathless to leave my home, or I get breathless when putting on or taking off my clothes

(6) Don't know

What other symptoms have you had, and how long did they last?

________________________________________________________________________________

________________________________________________________________________________

*(All participants* *were asked to complete the following section)*

Now there are some questions about your memory and concentration over the past 6 months.

Please select the answer that you feel best describes you (check one):

|  | Never | Sometimes | Often | Always |
| --- | --- | --- | --- | --- |
| Did you have difficulty remembering people's names? |  |  |  |  |
| Did you have trouble finding everyday objects (keys, glasses, wristwatch...)? |  |  |  |  |
| Did you find it difficult to remember situations that were important to you? |  |  |  |  |
| Did you find it difficult to concentrate when reading a book or newspaper? |  |  |  |  |
| Did you have difficulty completing what you started? |  |  |  |  |
| Did it take you longer than usual to perform daily tasks? |  |  |  |  |
| Did you ever feel disoriented on the street, as if you were lost? |  |  |  |  |
| Was it sometimes difficult for you to find the right words? |  |  |  |  |
| Were you easily distracted? |  |  |  |  |
| Did you have difficulty with simple mental calculations? |  |  |  |  |
| Did you feel like you couldn't keep up in a conversation (losing track)? |  |  |  |  |
| Did you notice it was challenging for you to learn new knowledge/information? |  |  |  |  |
| Did you have trouble maintaining focus on a specific task for an extended period? |  |  |  |  |

**Questionnaire – follow-up (6 and 12 months)**

*The specific month and/or year when the participant last answered a questionnaire was written in the section marked with "XX-XXXX” or “XXXX-XXXX”*

*(Introductory message to participants)*

Dear participant,

You previously took part in a survey on post-COVID-19 effects. We are reaching out to you again to inquire whether you would be willing to help us by responding to another questionnaire.

We would like to follow all participants for one year after the initial survey, regardless of whether they have had COVID-19 or not. This way, we can investigate whether individuals who have been infected with the coronavirus develop long-term effects, as well as the severity and duration of these symptoms. We are eager to compare all responses with those from people who have never had COVID-19, making every response equally important for the study.

We hope that you are willing to spend approximately 15-20 minutes answering the questionnaire below.

*(All participants were asked to complete the following sections)*

Please provide your full name

____________________________

Please provide your CPR number (personal Danish ID number)

____________________________

Compared to the last time you filled out a questionnaire in XX-XXXX how is your overall health now?

(1) Much better now than last time

(2) Somewhat better now than last time

(3) About the same

(4) Somewhat worse now than last time

(5) Much worse now than last time

Do you personally believe that you have had COVID-19 at any time since the last questionnaire in XX-XXXX?

(1) Yes

(2) No

(3) Don't know

If you answered yes to the last question - why do you think you may have had COVID-19?

Please check all that apply.

(1) I had symptoms and believe it was COVID-19

(2) I had symptoms and consulted a doctor who thought it was COVID-19

(3) I tested positive for coronavirus in a test (either self-test or PCR)

(4) Other reasons

Have you experienced any of the following issues with your work or other daily activities in the past 14 days due to your physical health?

|  | Yes | No |
| --- | --- | --- |
| I have reduced the time I spend on work or other activities |  |  |
| I have accomplished less than I would have liked |  |  |
| I have been limited in the type of work or other activities I could perform |  |  |
| I have had difficulty in performing my work or other activities (e.g., it required extra effort) |  |  |

Think about how you have felt in the last 14 days. Have you experienced any of the following issues?

|  | Yes | No | Don’t know |
| --- | --- | --- | --- |
| Concentration difficulties |  |  |  |
| Memory problems |  |  |  |
| Mental fatigue |  |  |  |
| Physical fatigue |  |  |  |
| Sleep problems |  |  |  |
| Fever or chills |  |  |  |
| Decreased or altered sense of taste |  |  |  |
| Decreased or altered sense of smell |  |  |  |
| Shortness of breath |  |  |  |
| Chest pain |  |  |  |
| Muscle and joint pain |  |  |  |
| Fatigue, exhaustion, or reduced muscle strength |  |  |  |

Have you experienced any other symptoms in the last 14 days? If yes, please specify.

________________________________________________________________________________________________________________________________________________________________

The following questions are answered only if you have selected yes to the corresponding symptom above.

How severe was your headache when you were at your worst?

(1) No pain

(2) Mild pain

(3) Moderate pain

(4) Severe pain

(5) Unbearable pain

How severe were your muscle or joint pains when you were at your worst?

(1) No pain

(2) Mild pain

(3) Moderate pain

(4) Severe pain

(5) Unbearable pain

How was your sense of taste different from normal?

My sense of taste was...

(1) Slightly impaired

(2) Significantly impaired

(3) Completely lost

(4) Altered

(5) Don't know

How was your sense of smell different from normal?

My sense of smell was...

(1) Slightly impaired

(2) Significantly impaired

(3) Completely lost

(4) Altered

(5) Don't know

Select below the statement(s) that best describe your breathing difficulties.

(1) I only get breathless when I exert myself greatly

(2) I get breathless when I hurry or walk up a slight hill

(3) I walk slower than others my age because of my breathlessness, or I have to stop to catch my breath when walking on level ground

(4) I stop to catch my breath after about 100 meters or a few minutes of walking

(5) I am too breathless to leave my home, or I get breathless when putting on or taking off my clothes

(6) Don't know

Think about the time before last winter (XXXX/XXXX). Were you generally (i.e., often or chronically) troubled by any of the following physical symptoms?

|  | Yes | No | Don’t know |
| --- | --- | --- | --- |
| Fever or chills |  |  |  |
| Decreased or altered sense of taste |  |  |  |
| Decreased or altered sense of smell |  |  |  |
| Shortness of breath |  |  |  |
| Chest pain |  |  |  |
| Muscle or joint pain |  |  |  |
| Fatigue, exhaustion, or reduced muscle strength |  |  |  |

The following questions are answered only if you have selected yes to the corresponding symptom above.

How severe was your headache when you were at your worst in the period before the winter of XXXX/XXXX?

(1) No pain

(2) Mild pain

(3) Moderate pain

(4) Severe pain

(5) Unbearable pain

How severe were your muscle or joint pains when you were at your worst in the period before the winter of XXXX/XXXX?

(1) No pain

(2) Mild pain

(3) Moderate pain

(4) Severe pain

(5) Unbearable pain

How was your sense of taste different from normal in the period before the winter of XXXX/XXXX?

My sense of taste was...

(1) Slightly impaired

(2) Significantly impaired

(3) Completely lost

(4) Altered

(5) Don't know

How was your sense of smell different from normal in the period before the winter of XXXX/XXXX?

My sense of smell was...

(1) Slightly impaired

(2) Significantly impaired

(3) Completely lost

(4) Altered

(5) Don't know

Select below the statement(s) that best describe your breathing difficulties in the period before the winter of XXXX/XXXX.

(1) I only get breathless when I exert myself greatly

(2) I get breathless when I hurry or walk up a slight hill

(3) I walk slower than others my age because of my breathlessness, or I have to stop to catch my breath when walking on level ground

(4) I stop to catch my breath after about 100 meters or a few minutes of walking

(5) I am too breathless to leave my home, or I get breathless when putting on or taking off my clothes

(6) Don't know

Think about the period since you answered the last questionnaire in XX-XXXX.

Have you been on sick leave since you filled out the last questionnaire in XX-XXXX?

(1) Yes

(2) No

(3) Prefer not to answer

The next four questions are only relevant if you answered 'yes' to the previous question. If you answered 'no', please proceed to the next section.

Were you on sick leave due to the after-effects/long-term effects of COVID-19?

(1) Yes

(2) No

(3) Partially

(4) Don't know

If you have been both fully and partially on sick leave during the period, please check both answers below.

(1) Fully on sick leave

(2) Partially on sick leave

How long have you been either fully and/or partially on sick leave since the last questionnaire?

(1) Less than 2 weeks

(2) 2 - 4 weeks

(3) More than 4 weeks but not the entire period

(4) I have been fully on sick leave for the entire period

Have you been on sick leave in the last 14 days?

(1) Yes

(2) No

(3) Prefer not to answer

Have you been diagnosed by a doctor with any of the following conditions since the winter of XXXX/XXXX?

|  | Yes | No |
| --- | --- | --- |
| Depression |  |  |
| Anxiety |  |  |
| PTSD (Post-traumatic stress disorder) |  |  |
| Chronic fatigue syndrome |  |  |
| Fibromyalgia |  |  |

We would like to hear about your general health. Have you been diagnosed with any of the following chronic diseases since the winter of XXXX/XXXX?

Please check all that apply.

(1) No, none

(2) Diabetes

(3) Asthma

(4) High blood pressure

(5) COPD or other chronic lung disease

(6) Chronic or frequent headaches, including migraines

(7) Other chronic diseases

(8) Prefer not to answer

Now there are some questions about your memory and concentration in the period since you last answered a questionnaire in XX-XXXX.

Please select the answer that you feel best describes you (check one):

|  | Never | Sometimes | Often | Always |
| --- | --- | --- | --- | --- |
| Did you have difficulty remembering people's names? |  |  |  |  |
| Did you have trouble finding everyday objects (keys, glasses, wristwatch...)? |  |  |  |  |
| Did you find it difficult to remember situations that were important to you? |  |  |  |  |
| Did you find it difficult to concentrate when reading a book or newspaper? |  |  |  |  |
| Did you have difficulty completing what you started? |  |  |  |  |
| Did it take you longer than usual to perform daily tasks? |  |  |  |  |
| Did you ever feel disoriented on the street, as if you were lost? |  |  |  |  |
| Was it sometimes difficult for you to find the right words? |  |  |  |  |
| Were you easily distracted? |  |  |  |  |
| Did you have difficulty with simple mental calculations? |  |  |  |  |
| Did you feel like you couldn't keep up in a conversation (losing track)? |  |  |  |  |
| Did you notice it was challenging for you to learn new knowledge/information? |  |  |  |  |
| Did you have trouble maintaining focus on a specific task for an extended period? |  |  |  |  |
